# Cell Signaling Pathways That Promote Radioresistance of Cancer Cells

**DOI:** 10.3390/diagnostics12030656

**Published:** 2022-03-08

**Authors:** Michel M. Ouellette, Sumin Zhou, Ying Yan

**Affiliations:** 1 Department of Internal Medicine, University of Nebraska Medical Center, Omaha, NE 68198, USA; mouellet@unmc.edu; 2 Department of Radiation Oncology, University of Nebraska Medical Center, Omaha, NE 68198, USA; szhou@unmc.edu

**Keywords:** radiation therapy, cell signaling pathways, cell cycle checkpoint, DNA repair, apoptosis, autophagy

## Abstract

Radiation therapy (RT) is a standard treatment for solid tumors and about 50% of patients with cancer, including pediatric cancer, receive RT. While RT has significantly improved the overall survival and quality of life of cancer patients, its efficacy has still been markedly limited by radioresistance in a significant number of cancer patients (intrinsic or acquired), resulting in failure of the RT control of the disease. Radiation eradicates cancer cells mainly by causing DNA damage. However, radiation also concomitantly activates multiple prosurvival signaling pathways, which include those mediated by ATM, ATR, AKT, ERK, and NF-κB that promote DNA damage checkpoint activation/DNA repair, autophagy induction, and/or inhibition of apoptosis. Furthermore, emerging data support the role of YAP signaling in promoting the intrinsic radioresistance of cancer cells, which occurs through its activation of the transcription of many essential genes that support cell survival, DNA repair, proliferation, and the stemness of cancer stem cells. Together, these signaling pathways protect cancer cells by reducing the magnitude of radiation-induced cytotoxicity and promoting radioresistance. Thus, targeting these prosurvival signaling pathways could potentially improve the radiosensitivity of cancer cells. In this review, we summarize the contribution of these pathways to the radioresistance of cancer cells.

## 1. Introduction

Radiation therapy (RT) is routinely used for cancer treatment, and more than fifty percent of patients with cancer, including pediatric cancer, receive RT as part of their treatment [1]. When combined with chemotherapy, termed chemoradiation, RT provides additional benefits, as shown by better disease control and a significant improvement of the survival of cancer patients [2,3,4]. Although RT contributes approximately 40% of curative cancer treatment, radioresistance (intrinsic or acquired) remains a major problem that impedes RT efficacy for cancer treatment [5,6,7,8,9]. Furthermore, no approaches are currently available for radiosensitizing cancer cells or for stratifying cancer patients based on their potential in receiving the benefit of RT. Thus, a clear understanding of the biochemical mechanisms that promote cancer cell survival in response to RT is anticipated to facilitate identifying therapeutic targets to improve the efficacy of RT.

**Figure 1:**
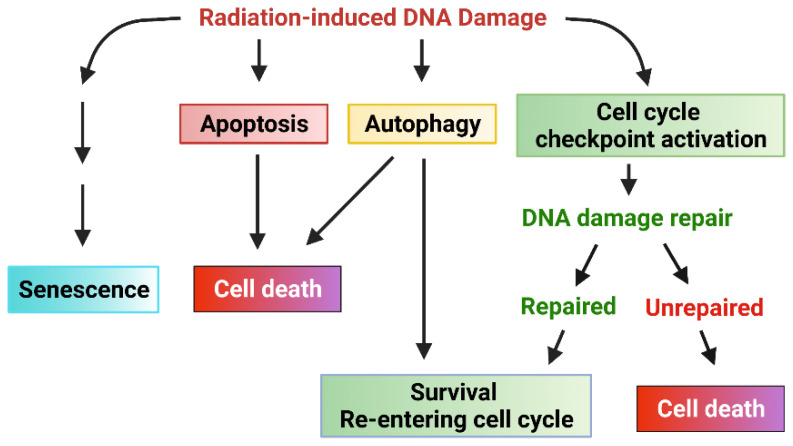
Cellular response to radiation-induced DNA damage. Ionizing radiation (IR) induces DNA damage in cancer cells in the form of either single-strand breaks (SSB) or double-strand breaks (DSB). DNA damage sensed by cells results in various cellular responses: senescence, apoptosis, autophagy, cell cycle arrest, and DNA repair. Signaling pathways that promote cell cycle checkpoint activation/DNA repair and inhibition of apoptosis can protect cancer cells from IR-induced cytotoxicity, promoting survival and the subsequent radiation resistance of cancer cells.

**Figure 2:**
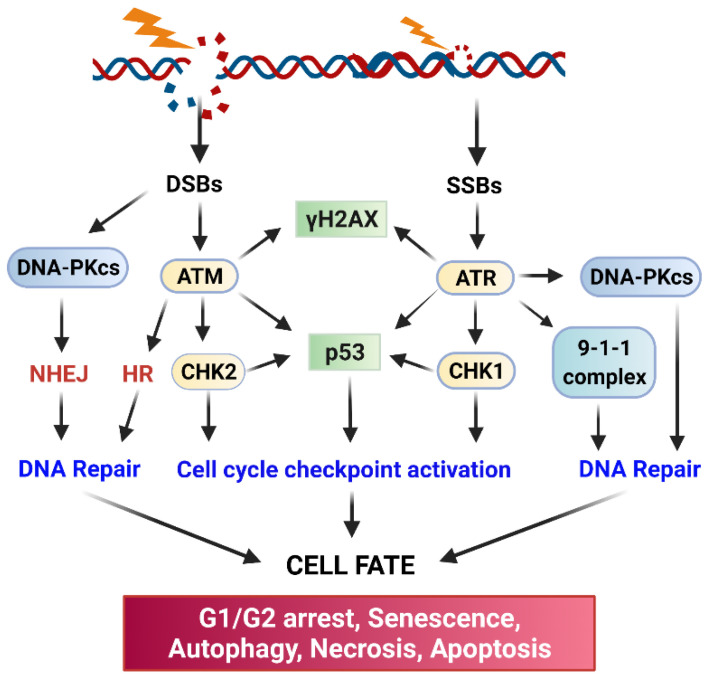
Core factors in DNA damage response and DNA repair networks. Ionizing radiation causes DNA damage that activates ATM, ATR, and DNA-PK kinases, which transmit signals to their downstream targets to promote DNA repair by NHEJ and HR while activating checkpoint response pathways to arrest the cell cycle. If the DNA damage cannot be repaired, other cellular signaling responses, such as those that lead to apoptosis, autophagy, and senescence induction will be triggered.

The current literature indicates that ionizing radiation (IR) can activate numerous cellular signaling pathways which lead to the induction of senescence, apoptosis, autophagy (leading to cell death or survival), and/or cell cycle checkpoint activation and DNA repair (Figure 1) [1,10,11,12,13,14,15]. With the latter, cells undergo cell cycle arrest to repair single- or double-stranded DNA damage with appropriate mechanisms and subsequently reenter the cell cycle if the damage is repaired. Accordingly, prosurvival signaling pathways in response to ionizing radiation (IR) are typically involved in the promotion of DNA repair and the inhibition of apoptosis induction [11,12,16,17] (Figure 2). These signaling pathways can act conjointly to minimize the radiation-induced cytotoxicity to cancer cells and, subsequently, biochemically reprogram the cancer cells to become radioresistant. In this review, we summarize the signaling pathways promoting cancer cell survival in response to IR.

## 2. IR-Induced DNA Damage Activates Cell Cycle Checkpoint Response to Promote Cell Cycle Arrest and DNA Repair

### 2.1. The Signaling Pathways That Activate Cell Cycle Checkpoint Response

The cytotoxicity caused by IR is mainly the result of DNA damage. IR induces several forms of DNA damage, which include single-stranded DNA breaks (SSBs), double-strand DNA breaks (DSBs), sugar and base damages, and DNA-protein crosslinks [12,17,18]. Among those, DSBs are the most lethal form of DNA damage, as misrepaired and unrepaired DSBs will result in genomic instability and cell death, respectively [19,20].

Upon DNA damage being sensed following IR, the cell cycle checkpoint will be activated to allow time for DNA repair [21]. Three cell cycle checkpoints exist, termed the G1 checkpoint, intra-S checkpoint, and G2 checkpoint, which block the cell cycle progression at the G1/S border, intra-S, or G2/M border, respectively, after detecting the DNA damage [21]. If the cell cycle checkpoints are defective in the cells or the DNA damage cannot be unrepaired, other responses (such as apoptosis, senescence, autophagy cell death, or necrosis) may be activated to eliminate the injured cells [21]. Therefore, a properly functional cell cycle checkpoint facilitates DNA repair in cancer cells, which is anticipated to promote the cell survival in response to IR.

ATM (Ataxia Telangiesctasia Mutated) and ATR (Ataxia Telangiectasia and Rad3-related) kinase-mediated signaling pathways play essential roles in the activation of cell cycle checkpoint response and DNA repair following radiation-induced DNA damage (Figure 2) [21,22]. Upon sensing DNA damage, ATM and ATR are rapidly activated, which, in turn, activate their downstream targets, including p53, DNA-activated protein kinase (DNA-PK), Checkpoint kinase (Chk)1, and Chk2 [21,22]. Activation of the Chk1/2 kinases results in the phosphorylation of Cell division control protein (Cdc)25 phosphatase, which leads to the subcellular sequestration (by 14-3-3), degradation, and/or inhibition of Cdc25 that otherwise activate the Cdk1 (Cyclin-dependent kinase 1)/Cyclin B activity to promote the G2/M transition of the cell cycle [23]. Furthermore, in response to IR, ATM, and ATR kinases, as well as Chk1 and Chk2 kinases, can directly phosphorylate and activate p53 tumor suppressor [21,22,24]. Consequently, activation of p53 by ATM, ATR, Chk1, and Chk2 results in a marked induction of p21 protein, which directly inhibits the activities of the Cdk4/Cyclin D, Cdk6/Cyclin D, and Cdk1/Cyclin A/B complexes to block the cell cycle progression [21,22].

Cell cycle progression requires the activities of Cdk kinases. While the G1/S transition of the cycle requires the activity of Cdk4/Cdk6 coupled with Cyclin D, the G2/M transition of the cell cycle requires the activity of Cdk1 coupled with Cyclin B [25,26]. The G1 checkpoint is mainly guarded by the p53 tumor suppressor and its transcriptional target p21, which directly binds to and inhibits Cdk4/6 [27]. The G2 checkpoint is controlled by the Cdk1/Cyclin B complex [26]. It is known that most cancer cells are defective in the G1 checkpoint due to the common mutations in the key regulators of the G1 checkpoint (e.g., p53, Cyclin D) [27]. However, most cancer cells possess a functional G2 checkpoint, which is operated mainly through p53-independent mechanisms [28]. Thus, abrogation of the G2 checkpoint in the cancer cells that are defective in the G1 checkpoint can sensitize the cells to radiation [29].

The inhibitory phosphorylation of Cdk1-Y15 by the Wee1 and Myt1 kinases inhibits Cdk1 activity, and it is the essential step for the activation of the G2 checkpoint by radiation [30]. Cdk1-Y15 resides in the ATP-binding domain of Cdk1, and phosphorylation of this site prevents the binding of ATP to Cdk1, thus inhibiting Cdk1 activity. The dephosphorylation of Cdk1-Y15 is catalyzed by the Cdc25 dual-specificity phosphatase that activates the Cdk1 activity [31,32,33]. During IR-induced G2/M cell cycle arrest, the phosphorylation of Cdk1-Y15 is maintained [30,34,35].

ATM, ATR, and DNA-PK also serve as major activators of DNA repair, and each of them is recruited to the DNA damage sites by a specific co-factor: Nijmegen breakage syndrome 1 (NBS1) (a component of the MRE11-RAD50-NBS1 complex) for ATM [36,37,38], ATRIP for ATR [39], and Ku80 for DNA-PKcs [40,41]. The initiation step will trigger the subsequent recruitment of additional co-factors required for the assembly of DNA repair apparatus at the sites of DNA damage. biochemically, DNA-PK primarily triggers DSB repair via non-homologous end-joining repair (NHEJ), ATM triggers both NHEJ and homologous recombination (HR) repair of DSB, and ATR mainly triggers HR-mediated DSB repair [22,42]. In addition, there is functional redundancy and crosstalk among the three DNA damage response (DDR) pathways. Ultimately, activation of the three pathways by radiation results in the inhibition of Cdk activities leading to cell cycle arrest to allow time for DNA repair and cell survival [12].

### 2.2. DNA Repair Pathways

In response to the DNA damage by IR, cancer cells rapidly activate ATM, ATR, and DNA-PK, all of which are members of the phosphoinositide 3 kinase-related kinase family. These kinases transduce the DNA damage signaling, coordinate the assembly of DNA repairing apparatuses at the damaged sites and initiate the repairing of DNA (Figure 2) [17]. IR-induced DSBs are repaired mainly either by NHEJ or HR [17]. NHEJ directly re-ligates the free-ends of the broken DNA without the need for a homologous template and, thus, it is an error-prone process [43]. To begin, NHEJ first requires the recruiting of the Ku70/Ku80 heterodimer to each end of the broken DNA and the formed complex triggers subsequent recruiting of DNA-PKcs that results in the juxtaposition of the two DNA ends. The Ku70/Ku80/DNA-PKcs complex further recruits the DNA ligase complex (XRCC4/XLF/DNA ligase IV/PNK) to process the final ligation [43]. In contrast to NHEJ, HR takes advantage of sequence information present in the intact sister chromatid, accurately repairing DSBs with high fidelity [43]. Thus, since NHEJ does not require a DNA template for the repair, it can function through the cell cycle. In contrast, HR mainly operates during the S and G2 phases when a DNA template becomes available after the DNA replication [43]. Radiation also produces SSBs, which are mainly caused by base oxidation by ROS/RNS [19]. To repair this type of damage, the cell uses the base excision repairing mechanism. To process the repair, the single and multiple damaged bases will first be removed by DNA glycosylase-mediated incision and apurinic endonuclease 1 (APE1)-mediated incision, respectively, and the generated nicks will be filled up by the joint work of DNA polymerases and the DNA ligase [44]. In the end, the successful repair of the damaged DNA caused by IR permits cells to survive and reenter the cell cycle. On contrary, failure of repairing the damaged DNA will result in one of the following outcomes: senescence, autophagy, necrosis, or apoptosis (Figure 2).

## 3. Radiation Induces Several Prosurvival Signaling Pathways

### 3.1. Epidermal Growth Factor Receptor (EGFR) Tyrosine Kinases Mediate Prosurvival Signaling Pathways in Response to IR

**Figure 3:**
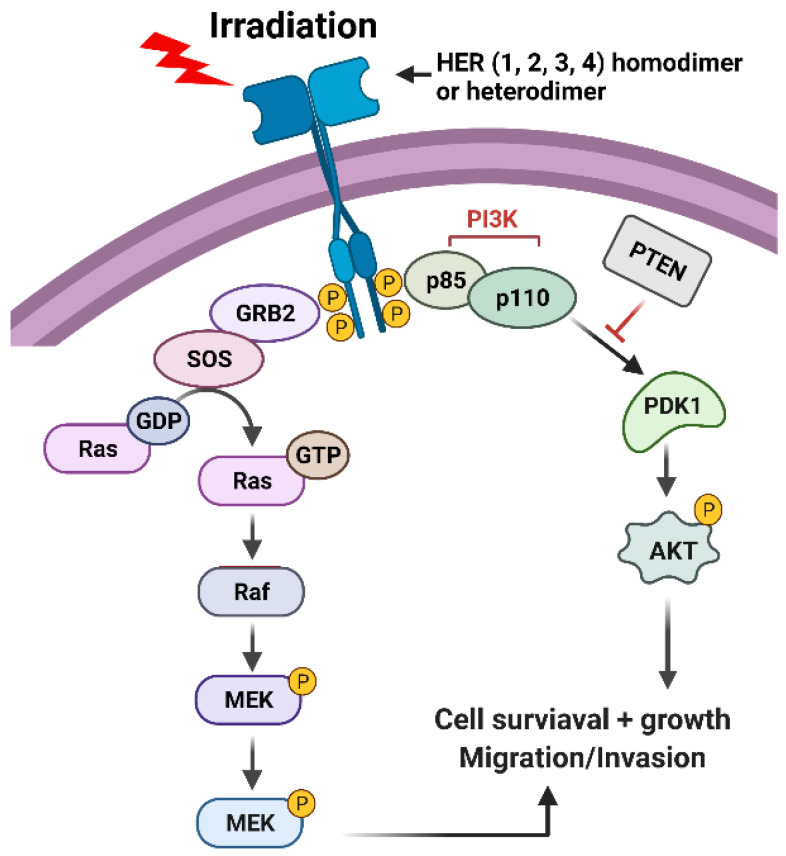
Radiation induces activation of HER receptors, which, in turn, leads to the activation of PI3K/AKT and RAS/RAF/MEK/ERK signaling pathways that promote cell survival.

The EGFR/epidermal growth factor receptor (ErbB)/HER family of receptor tyrosine kinases (RTKs) are trans-membrane proteins that share a similar structure, which contains an extracellular region, a transmembrane region, and an intracellular region (Figure 3) [45,46]. There are four members in the family, EGFR/HER1, ErbB2/HER2, ErbB3/HER3, and ErbB4/HER4 (Figure 3) [45,46]. While the extracellular region contains the ligand binding and dimerization domains, the intracellular region contains the tyrosine kinase domain and phosphorylation regulatory tail [47]. Among them, ErbB2/HER2 does not bind to any ligand, and ErbB3/HER3 exhibits a very low enzymatic activity [47]. As a result, a ligand can only bind to EGFR/HER or ErbB4/HER4, which stimulates either the homo- or hetero-dimerization of the RTK receptors, and the subsequent trans-phosphorylation of the c-terminal regulatory tail of the receptors (Figure 3) [47]. Afterward, the phosphorylated tyrosine residues form docking sites for downstream adaptors and signal transducers, triggering the activation of downstream signaling pathways such as PI3K (phosphoinositide 3-kinases)/AKT (AKT8 virus oncogene cellular homolog), RAS/RAF/MEK (MAPK/Erk kinase)/ERK (Extracellular signal-regulated kinase), phospholipase C-γ/protein kinase C and JAK (Janus-family tyrosine kinase)/STAT (Signal transducer and activator of transcription) pathways [46,48]. Among those, PI3K/AKT and RAS/RAF/MEK/ERK signalings play significant roles in promoting cell survival following irradiation (Figure 3) [49,50].

It has been reported that IR exposure induces EGFR (Epidermal Growth Factor Receptor)/HER1 (Human epidermal growth factor receptor 1) phosphorylation, indicative of its activation [51,52,53]. Our work with human breast cancer cells demonstrates that IR activates not only the phosphorylation of HER1 but also the phosphorylation of HER2, HER3, and HER4 [54]. Although the mechanism causing this effect of IR has not yet been clearly elucidated, it might be attributed to the inhibition of the receptor protein tyrosine phosphatases (PTPs) that dephosphorylate the HER RTKs. The evidence supporting this concept is that receptor PTPs can be efficiently inactivated by reactive oxygen/nitrogen species (ROS/RNS) through the oxidation of their enzymatic active sites such as those that contain Cysteine residue [55], and IR has been shown to induce ROS/RNS production via a mitochondria-dependent mechanism [56]. Thus, IR-induced ROS/RNS could inhibit the membrane-bound receptor PTPs, and, in turn, result in the activation of HER RTKs.

ErbB/HER RTKs have been implicated in promoting cancer cell survival in response to radiation, which is likely to involve the following two mechanisms: (1) inducing the prosurvival AKT and ERK1/2 signaling pathways [45,46] (Figure 3), and (2) facilitating cell cycle checkpoint response to promote DNA repair [49,50]. Supporting the concept, studies from our group have shown that HER2 activation is necessary for the induction of the G2/M checkpoint following IR in breast cancer cells [54], and others have demonstrated a role of EGFR/HER1 in promoting the activation of DNA-PK that is essential for initiating DSB repair by NHEJ [57,58].

### 3.2. Ras-Related C3 Botulinum Toxin Substrate 1 (Rac1) Mediated Signaling Pathways in IR Response

Rac1 belongs to the Rho family of GTPases that play key roles in cytoskeleton reorganization, cell polarity, and cell migration [59]. Like all GTPases, Rac1 is active in its GTP-bound form and inactive in its GDP-bound form [60]. The exchange of GDP to GTP on Rac1 is facilitated by its GEFs (Guanine nucleotide Exchange Factors), while the hydrolysis of GTP on Rac1 is promoted by its GAPs (Guanine nucleotide Exchange Factors) [60]. Upon activation, Rac1 transduces numerous downstream signaling pathways [61,62]. Through its downstream effector PAK1/2 kinases that phosphorylate/activate Raf1 and MEK1 kinases, Rac1 can induce the activation of the extracellular signal-regulated kinase (ERK1/2) mediated signaling that promotes cell survival and proliferation [63,64,65]. Similarly, Rac1 via PI3K can activate the AKT signaling pathway that plays a significant role in cell survival in response to various stimuli [66,67,68]. Both AKT and ERK1/2 signalings promote survival following IR exposure [69,70,71,72,73,74,75].

Our studies have revealed the role of Rac1 in promoting the survival of breast and pancreatic cancer cells responding to IR exposure [76,77,78]. Our data indicate that IR induces a rapid activation Rac1 activity, which is essential for IR-induced ATM/ATR signalings that lead to G2 checkpoint activation and cell survival. Our studies also show that Rac1 activity is required for the resistance of breast cancer cells to the clinical protocol of hyper fractionated radiation treatment and the upregulation of the expression of Bcl-xL anti-apoptotic protein in the RT-resistant cells [78]. Consistently, studies by others show that deficiency in Rac1 function attenuates cell cycle checkpoint response, DNA repair, and cell survival in response to both IR and UV irradiation [79].

### 3.3. IR-Activated ERK1/2 Signaling Pathway

ERK1/2 signaling activation in response to IR is commonly observed in cancer cells, and evidence suggests at least four mechanisms contributing to this biological event. The first mechanism involves the activation of the ErbB/HER receptors by IR. As discussed above, we and others have demonstrated an essential contribution of ErbB/HER RTKs to the activation of ERK1/2 signaling in breast and lung cancer cells in response to IR (Figure 3) [52,54]. In addition, Ras activation by ErbB/HER receptors induces EGFR-ligand production, resulting in an autocrine feedback loop that can further enhance the Ras/Raf/MEK/ERK signaling cascade [80,81]. Consistent with the finding, ectopic expression of Ras-N17 dominant-negative mutant, which inhibits the endogenous Ras GTPase, abrogates the IR-induced ERK1/2 signaling activation [82,83]. The second mechanism involves the BRCA1 tumor suppressor. Our studies demonstrate that BRCA1 protein expression is required for the IR-induced activation of ERK1/2 signaling in breast cancer cells, and conversely, ERK1/2 activity supports the protein stability of BRCA1 in the irradiated breast cancer cells [72]. These results suggest a positive feedback loop regulation between ERK1/2 signaling and BRCA1 protein stability in response to IR, which may play an important role in sustaining the G2/M cell cycle checkpoint response following IR, as inhibition of either BRCA1 or ERK1/2 in breast cancer cells abolishes G2/M cell cycle arrest and results in a concomitant induction of apoptosis [72]. The third mechanism involves the ATM kinase. It has been shown that inhibition of ATM partially blocks the induction of ERK1/2 signaling following IR, and, likewise, inhibition of ERK1/2 attenuates radiation-induced ATM phosphorylation, as well as the recruitment of ATM to DNA damage foci [84]. This displays another positive feedback loop in the radiation response, this time involving ATM and ERK1/2 signalings. The fourth mechanism involves the Rac1-GTPase signaling. As discussed above, IR induces a rapid Rac1 activation, which, in turn, through its downstream effector PAK1/2 kinases, activates the Raf/MEK/ERK signaling [63,64,65].

The main function of ERK1/2 signaling activation by IR is to promote cell survival [69,70,71,72]. ERK1/2 signaling activates many transcription factors that increase the expression of the genes encoding for anti-apoptotic proteins [85,86]. The best-known anti-apoptotic transcription factors that are activated by ERK1/2 in response to IR include CREB (cyclic AMP-responsive element-binding protein) and C/EBP-β (CAAT/enhancer-binding protein β), both of which are induced by p90rsk that is directly substrate of ERK1/2 kinases. The activated CREB and C/EBP-β, in turn, induce the expression of several anti-apoptotic proteins such as B cell leukemia (Bcl)-xL, Myeloid cell leukemia (Mcl)-1, and c-FLICE inhibitory protein (FLIP)s [87,88,89]. Furthermore, ERK1/2 directly inhibits several pro-apoptotic proteins that include Bad, Bim, and caspase 9 via inhibitory phosphorylation [90,91,92,93].

ERK1/2 signaling has also been shown to promote DNA repair in response to IR. We and others have demonstrated an essential role for ERK1/2 signaling in the activation of the G2/M DNA damage checkpoint in response to IR, and this involves the ERK1/2 activity in the activation of ATR and BRCA1, both of which are key regulators of the G2 checkpoint response and DNA repair [69,71,72,94,95]. Furthermore, IR-induced ERK1/2 signaling has also been linked to the transcriptional up-regulation of the genes involved in DNA repairs, such as excision repair cross complementation group 1(ERCC1), X-ray repair cross-complementing group 1 (XRCC1), and Xeroderma pigmentosum complementation group C (XPC) [96,97]. Moreover, ERK1/2 activates DNA-PK, which is required for NHEJ-mediated DSB repair, and PARP-1, which is essential for repairing SSBs [97,98,99,100]. In addition, ERK1/2 signaling positively regulates ATM-dependent HR for DSB repair [84]. Thus, the positive role of ERK1/2 signaling in cancer cell survival following radiation is also through its promotion of G2/M checkpoint activation and DNA repair. Consistent with these observations, several studies demonstrate that constitutive activation of Ras increases the radioresistance of cancer cells, whereas inhibition of MEK or ERK leads to the radiosensitization of cancer cells [69,76,94,95].

Collectively, the activation of ERK1/2 signaling by IR involves multiple mechanisms. In return, ERK1/2 activation promotes cell survival by both promoting DNA repair and blocking apoptosis induction.

### 3.4. The PI3K/AKT Signaling Promotes Cell Survival in Response to IR

**Figure 4:**
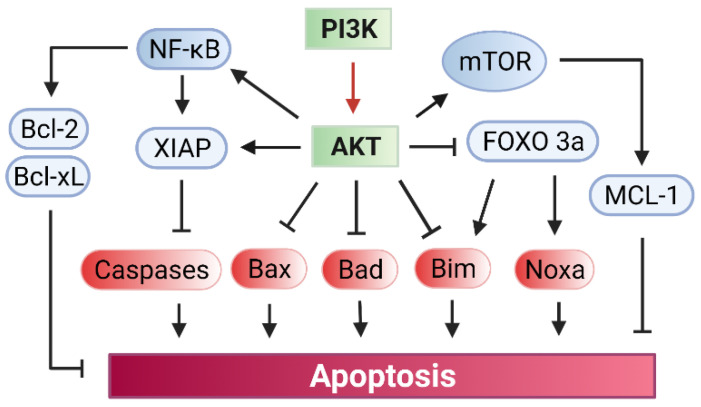
PI3K/AKT mediated signaling promotes cell survival. (i) Activation of PI3K by radiation leads to the phosphorylation/activation of AKT; (ii) AKT phosphorylates and inhibits the pro-apoptotic proteins Bad, Bax, and Bim; (iii) AKT activates Nuclear factor (NF)-κB transcription factor, resulting in the up-regulation of the expression of pro-survival genes Bcl-2 and Bcl-xL; (iv) AKT phosphorylates the pro-survival protein XIAP, which binds to and inhibits caspase3/7/9 that are required for apoptosis induction; (v) AKT phosphorylates/activates the mammalian target of rapamycin (mTOR) kinase, which then phosphorylates and activates the anti-apoptotic protein Mcl-1; (vi) phosphorylation of Forkhead box O (FOXO)3a (transcription factor) by AKT results in the inhibition and nuclei exclusion of FOXO3a, which otherwise up-regulates the gene expression of pro-apoptotic proteins Bim and Noxa.

The PI3K/AKT signaling plays a critical role in blocking apoptosis induction, which relies on the AKT function in the direct inhibition of several pro-apoptotic proteins, while upregulating several anti-apoptotic pathways (Figure 4) [101,102,103,104].

By phosphorylation, AKT can directly inhibit the key pro-apoptotic proteins Bad, Bax, and Bim, all of which are members of the Bcl-2 family (Figure 4) [102,103,104]. Furthermore, AKT phosphorylates the transcription factor FOXO3a (Forkhead box O3), resulting in the cytoplasmic retention and subsequent proteasomal degradation of FOXO3a, which otherwise increases the expression of pro-apoptotic factors Bim and Noxa to promote apoptosis [105,106,107,108].

PI3K/AKT signaling also promotes several anti-apoptotic pathways, which include the pathways of NF-κB, XIAP, and mTOR (Figure 4). Firstly, as discussed below in the NF-κB section, PI3K/AKT signaling activates the NF-κB transcription factor by freeing it from the bound inhibitor IκB, allowing NF-κB to translocate into the nuclei to induce expression of a variety of anti-apoptotic genes, especially Bcl-2 and Bcl-xL [109]. Secondly, AKT phosphorylates/activates XIAP (X-linked inhibitor of apoptosis protein), which then binds to and inactivates the caspases 3, 7, and 9 that are required for apoptosis induction [110]. Thirdly, AKT phosphorylates and activates mTOR kinase, which, in turn, phosphorylates and activates Mcl-1 anti-apoptotic protein [111,112]. Fourthly, AKT directly phosphorylates and activates the catalytic subunit of DNA-PK, which is the driver of the NHEJ repair of DSB that promotes cell survival in response to IR [113].

AKT has been shown to negatively regulate apoptosis induction by hypoxia, a condition that is often produced by radiation therapy [114,115]. It has been shown that GSK3 (glycogen synthase kinase 3) plays a central role in triggering hypoxia-induced apoptosis through its activation of the mitochondria-dependent death-signaling pathway [115,116]. However, AKT can inhibit GSK3 by inhibitory phosphorylation at the Ser 9, which results in the activation of glycolysis and glucose transport that inhibit apoptosis induction by hypoxia [117].

Activation of the PI3K/AKT signaling following IR has been frequently detected in cancer [49,50]. As discussed above (Figure 3), the most likely mechanism involves the activation of ErbB/HER receptors by IR, as the phosphorylation of the carboxyl-terminal regulatory tail of ErbB3/HER3 produces six docking sites for the binding of the p85 adaptor subunit of PI3K (Figure 3) that phosphorylates PIP2 (phosphatidylinositol-4,5-biphosphate) to generate PIP3 (phosphatidylinositol (3,4,5)-triphosphate), which results in the recruitment and activation of PDK1 (phosphoinositide-dependent kinase 1) [118,119]. Upon activation, PDK1 phosphorylates AKT-Thr308 to partially activate AKT, which primes the further phosphorylation of AKT-Ser473 by PDK2 that fully activates AKT activity [119]. In addition, Ras activation by ErbB/HER receptors or via mutations can also positively regulate the IR-induced PI3K/AKT signaling through its activation of the production of EGFR ligands that further activate the ErbB/HER signaling [120,121].

Collectively, the pro-survival function of PI3K/AKT signaling is predicted to promote the radioresistance of cancer cells and the concept has also been supported by numerous studies both in vitro and in vivo. These studies show that inhibition of PI3K/AKT signaling either by chemical or biological inhibitors can enhance the radiosensitivity in some cancer cell types, which is accompanied by diminished DNA repair and increased apoptosis induction [73,74,75,113,122,123]. However, inhibition of PI3K/AKT in some cell models shows little effect on radiosensitivity [27,69,124,125,126]. These studies indicate that the effect of PI3K/AKT signaling on the radiosensitivity of cancer cells is probably cell-type specific.

### 3.5. NF-κB Signaling Pathway Promotes Radioresistance

**Figure 5:**
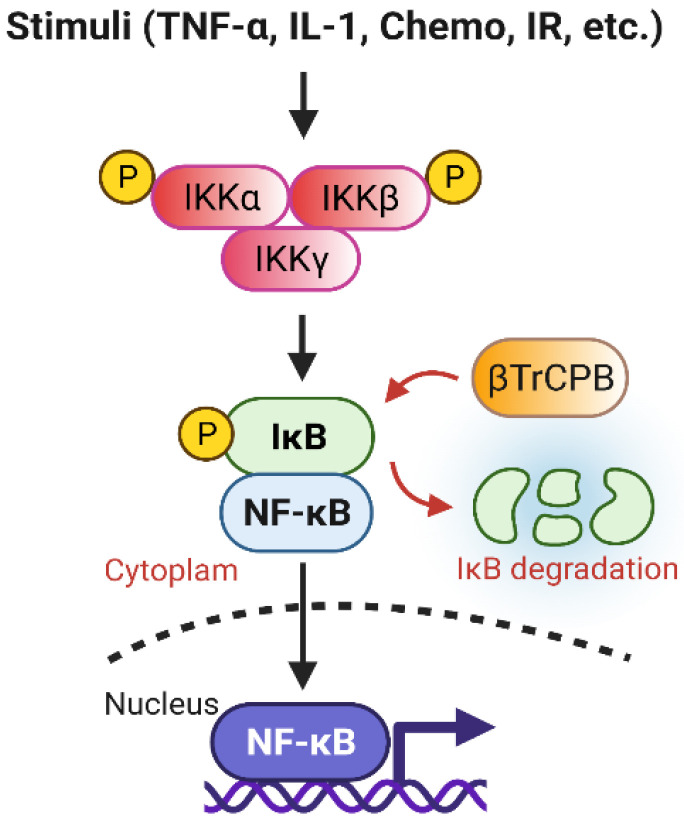
Overview of the NF-κB signaling pathway. NF-κB is inhibited by IκB in the cytoplasm. Upon activation by upstream signals (e.g., Tumor Necrosis Factor (TNF)-α, Interleukin (IL)-1, Chemo, IR), IκK (IκB kinase) phosphorylates IκB, resulting in its proteasomal degradation by the Skp_Cullin_F-box (SCF)^βTRCP^–ubiquitin ligase complex. Consequently, this frees NF-κB, allowing it to be translocated into the nucleus to activate gene transcriptions that promote proliferation and survival.

NF-κB, a heterodimer consisting of p50 and RelA, is a transcription factor playing an important role in the regulation of inflammatory response to various stimuli including radiation and chemotherapy drugs (Figure 5) [127,128,129]. NF-κB normally is inhibited by the Inhibitory κB protein (IκB) that sequesters NF-κB in the cytoplasm [129]. Following stimulation, activated IκKs phosphorylate IκB, resulting in its degradation promoted by βTrCP [129]. This releases the sequestered NF-κB, which then translocates into the nuclei and induces expressions of its target genes that promote survival and proliferation [129]. Additionally, IR-induced ATM and ROS can further enhance the NF-κB pathway [130]. The best validated antiapoptotic gene targets of NF-κB are Bcl-2, Bcl-xL, and Mcl-1, which are members of the Bcl-2 family. Furthermore, IR activates NF-κB to express cell cycle-specific genes, such as cyclin D1, which is also implicated in radioresistance [128]. Consequently, hyperactivity of the NF-κB signaling has been linked to the radioresistance of cancer cells.

## 4. Radiation Activates the Autophagy Signaling Pathway That Leads to Either Cell Survival or Cell Death

Autophagy is a highly programmed process of lysosome-mediated degradation, which is a conserved cellular defensive mechanism against various stress stimuli such as oxidation, nutrient deprivation, ER stress, and DNA damage [131,132]. The mTOR kinase plays a central role in the regulation of autophagy induction, as its activation by AKT and MAPK inhibit autophagy induction while its inhibition by AMPK and p53 promotes autophagy induction [131,132,133]. The activation of autophagy signaling begins with the inhibition of mTOR kinase and activation of ULK kinase, which subsequently complexes with and activates ATG13 and FIP200. Meanwhile, PI3K-III forms another complex with the other autophagy-related proteins (ATG14, VPS24, Beclin1, and p150). Both the PI3KIII complex and ULK complex are then recruited to a double-layer membrane structure to form phagophores that ultimately fuse with lysosomes and proceed to the degradation of the protein cargos [132,133]. Generally, the function of autophagy is thought to promote cell survival through maintaining energetic homeostasis. However, autophagy can also induce cell death to eliminate the seriously damaged cells [131,132,134]. It has been known that radiation-produced ROS/RNS not only causes oxidative stress and impedes mitochondrial function in cells but also induces DSB/SSB DNA damage, all of which can trigger autophagic response leading to cell death or survival. The latter protects the irradiated cancer cells, thus promoting radioresistance [5,133,135,136,137]. Multiple factors can also affect the fate of the autophagy response to radiation. These include the cell types, the degree of damage, and/or nutrient conditions [14,138,139].

## 5. HIF-1α Signaling Pathway Facilitates Radio-Protective Mechanisms in Tumor Cells

The current literature supports an important role of hypoxia-inducible factor-1 (HIF-1), a transcription factor that serves as a master regulator of cellular responses to hypoxia, in the promotion of radioresistance of tumor cells under both hypoxia and normoxia conditions [140].

HIF-1 is a heterodimer consisting of one hypoxia-inducible subunit (HIF-1α, HIF-2α or HIF-3α) and HIF1β that is constantly expressed and insensitive to the cellular oxygen concentration [140,141]. HIF-1α and HIF-2α, which share 48% identity in their protein sequences, have been shown to contribute to tumor resistance to radiation therapy [141]. While HIF-1α is ubiquitously expressed in almost all cell types and tissues, HIF-2α is mostly expressed only in the vascular endothelium, lung and heart tissues, and placenta [142].

**Figure 6:**
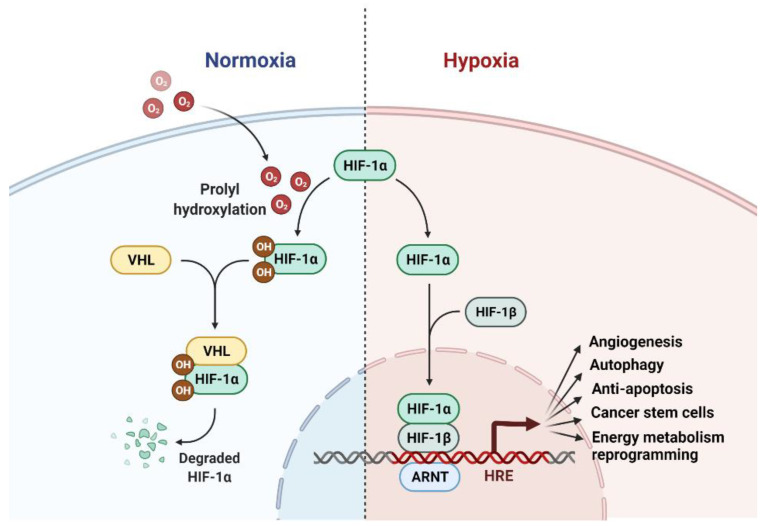
Overview of the hypoxia-inducible factor 1 (HIF-1) signaling pathway under normoxia and hypoxia conditions. Under normoxia, HIF-1α is hydroxylated by prolyl-4-hydroxylase, leading to its interaction with von Hippel-Lindau (VHL) and subsequent proteasomal degradation. Under hypoxia, HIF-1α accumulates and translocates to the nucleus and forms a complex with HIF-1β. The heterodimer complexes then bind to the hypoxia response element (HRE) with p300/CBP and activate the expression of their targeted genes, which include those that promote angiogenesis (e.g., VEGF), autophagy (e.g., LC3-II), anti-apoptosis (e.g., BCL-2), cancer stem cell stemness (e.g., Snail), and energy metabolism reprogramming e.g., GLUT1).

Under normoxia, the ODDD (oxygen-dependent degradation domain) domain of HIF1α are hydroxylated at Pro-402 and Pro-564 residues by the α-ketoglutarate- and O2-dependent prolyl-4-hydroxylases, resulting in the proteasomal degradation of HIF1α that are mediated by the von Hippel-Lindau (VHL) E3 ubiquitin ligase (Figure 6). In contrast, under hypoxia, HIF-1α becomes stabilized and translocates into the nuclei to form a complex with HIF-1β and followed by recruiting the transcriptional adapter/histone acetyltransferase proteins, p300 and CBP (CREB-binding protein), to a transcription complex that activates the transcription of HIF-1 targeted genes [143]. In addition to the primary regulation by VHL, HIF1α expression can also be regulated by STAT3 (Signal Transducer and Activator of Transcription 3), NF-κB nuclear factor-κB, microRNAs/long noncoding RNAs, c-Myc, angiotensin II, and signaling pathways involving stress- or the mitogen-activated kinases, PI3K and mTOR [141,144,145].

While the tumor microenvironment is generally more hypoxic than the surrounding normal tissue, which is attributed to the rapid expansion of tumor volume versus the relatively delayed growth of blood vessels that supply oxygen, most tumor cells are defective in the mitochondrial oxidative energy metabolism but using glycolysis as the main energy metabolic pathway, known as the Warburg effect [140]. As a result, the antioxidants (NADPH and glutathione) produced by glycolysis can efficiently absorb the ROS produced by IR in cancer cells, resulting in the stabilization of HIF-1α and its radioprotective effect in tumor cells under normoxia [145,146].

The ability of HIF-1 to promote the radioresistance of tumor cells is through its activation of multiple radioprotective mechanisms including angiogenesis, autophagy, inhibition of apoptosis, supporting cancer stem cell (CSC) stemness, and reprogramming energy metabolic pathways (Figure 6): (1) HIF-1α promotes angiogenesis by upregulation of VEGF (vascular endothelial growth factor) expression and increases anaerobic glycolysis [140]; (2) HIF-1α has been shown to promote autophagy induction in hypoxic cancer cells via increasing the expression of beclin and LC3-II, which are key components of the autophagic pathway, and enhancing the miR-210/Bcl-2 and Akt/mTOR/P70S6K pathways; (3) HIF-1α has been shown to promote cancer cell survival in response to radiation. This role involves the HIF-1α’s ability to diminish ROS production (as described above) and increase the expression of microRNA 210, which modulates its mRNA targets to promote DNA repair, autophagy induction, and apoptosis inhibition [147]. In return, HIF1α-induced miR-210 through a positive feedback loop further stabilizes HIF-1α and enhances its positive impact on radioresistance of hypoxic tumors; (4) Cancer stem cells (CSCs) are known to be radioresistant. HIF1α induces the transcription of many genes that are essential for the maintenance of CSC stemness [141,144]. Such genes include those involved in survival, self-renewal (e.g., hTERT, ABC-Ts, Notch), and promoting the EMT (Epithelial-mesenchymal transition) phenotype (e.g., TAZ, Snail, Twist, Slug, Zeb-1/2); and (5) HIF-1α plays a central role in the hypoxia-activated reprogramming of the energy metabolism in cancer cells. HIF-1α induces the expression of the key enzymes and regulators of glucose metabolism (such as glucose transporter 1 (Glut1)), resulting in the Warburg effect, which is resulted by the shift of the ATP-generating pathway from the more efficient mitochondrial oxidative phosphorylation to less efficient glycolysis [145,146]. Consequently, such a shift results in a marked decrease in ROS production and an intracellular accumulation of reduced glutathione (GSH), both of which can effectively diminish the radiosensitivity of hypoxic tumor cells, leading to radioresistance (Figure 6). Collectively, HIF-1α participates in the regulation of multiple radioprotective mechanisms in hypoxic tumors and, thus, the inhibition of the HIF-1α involved pathways may be targeted for radiosensitization of tumor cells.

## 6. YAP Signaling Pathway Promotes Radioresistance of Tumors

**Figure 7:**
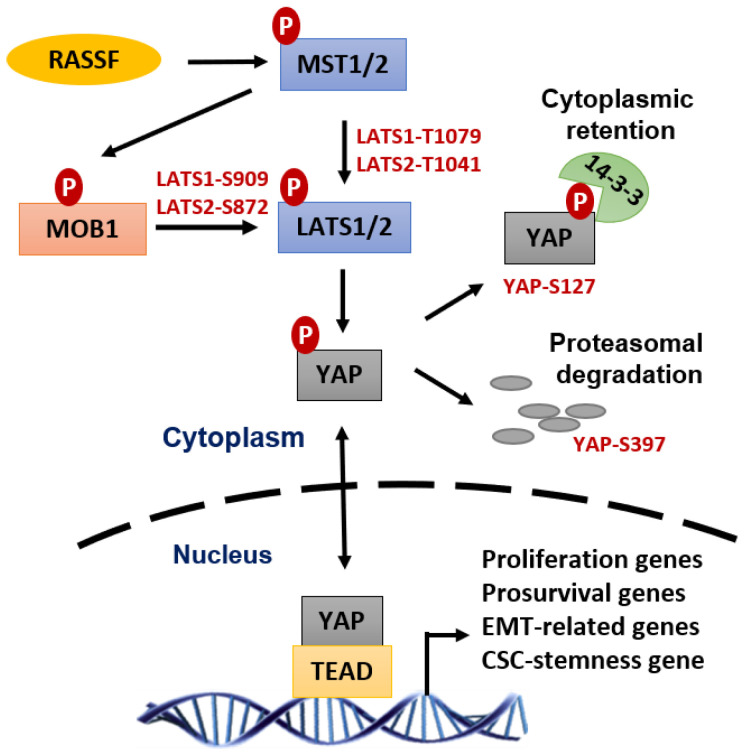
Yes-associated protein 1 (YAP) transcriptional activity promotes radiation-therapy resistance. As a master transcriptional coactivator of TEA Domain Transcription Factor (TEAD), YAP activates the transcription of many essential genes that support survival, DNA repair, proliferation, and epithelial-mesenchymal transition (all of which can synergistically drive cancer cells resilient to radiation-induced cytotoxicity and developing radioresistance.

Yes-associated protein (YAP) is a transcription coactivator of the Transcriptional Enhanced Associate Domain (TEAD) family of transcription factors. YAP activates the transcription of many genes required for tumorigenesis and metastasis of most solid tumors [148,149,150,151]. YAP is inhibited by the Hippo tumor suppressor pathway, whose activation results in the phosphorylation of YAP at multiple sites, leading to the cytoplasmic retention of YAP by 14-3-3, and proteasomal degradation of YAP promoted by the βTrCP-SCF ubiquitin ligase complex [152] (Figure 7).

YAP activation has been positively linked to the intrinsic radioresistance of several cancer types, including cancer of the brain (Glioblastoma and medulloblastoma), breast, lung, and pancreas [151,153,154,155,156]. While the detailed mechanisms that lie beneath this YAP effect remain to be clearly delineated, previous studies suggest that it generally involves the YAP function in the transactivation of gene expressions required for cell survival (Survivin, Bcl-2/Bcl-XL, etc.), DNA repair (p73, etc.), proliferation (EGFR/HER, Axl, cell cycle genes, MAPK, etc.), and cancer stem cells (SOX2, CTGF, Cyr61, etc.) [151,157]. While the detailed mechanisms that lie beneath this YAP effect remain to be clearly delineated, previous studies suggest that it generally involves the YAP function in the transactivation of gene expressions required for cell survival (Survivin, Bcl-2/Bcl-XL, etc.), DNA repair (p73, etc.), proliferation (EGFR/HER, Axl, cell cycle genes, MAPK, etc.), and cancer stem cells (SOX2, CTGF, Cyr61, etc.) [151,152]. Accordingly, these YAP-promoted prosurvival pathways are predicted can conjointly reduce radiation-induced cytotoxicity and promote the radioresistance of cancer cells through facilitating DNA repair, inhibiting apoptosis, and preserving cancer stem cells.

## 7. Conclusions

**Figure 8:**
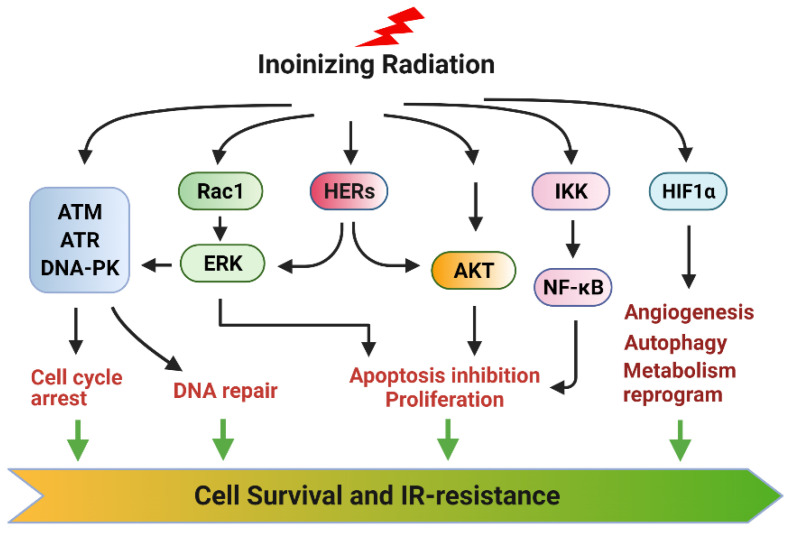
Overview of ionizing radiation-induced signaling pathways that promote tumor cell survival and radiation resistance. Activation of ATM, ATR, and DNA-PK signalings by radiation leads to cell cycle arrest and DNA repair. Activation of HER, ERK1/2, and AKT signaling pathways by radiation inhibits apoptosis induction and promotes cell cycle checkpoint response and DNA repair. Antioxidants produced by glycolysis reduce ROS levels in tumor cells to sustain HIF-1α activity that promotes radioprotective mechanisms.

RT is a standard approach for cancer treatment, whereas radioresistance (intrinsic or acquired) has remained a significant clinical problem that limits the efficacy of RT. A significant challenge is that the radiation concomitantly activates multiple prosurvival signaling pathways that block apoptosis induction, promote DNA repair and adaptive energy metabolic changes, and induce angiogenesis, which together markedly reduce the magnitude of RT-induced lethality in cancer cells. Among those, the main function of AKT, ERK1/2, and NF-κB signalings is to block apoptosis induction in the irradiated cancer cells, while the primary functions of the ATM, ATR, and DNA-PK signalings are to promote the cell cycle checkpoint activation and DNA repair in cancer cells. In addition, ERK1/2 and AKT signalings also positively regulate the cell cycle checkpoint response and facilitate DNA repair. Furthermore, hypoxia-activated HIF-1α promotes angiogenesis, autophagy, CSCs, and reprograming energy metabolism to diminish radiation damage in cancer cells. Moreover, YAP signaling contributes to intrinsic radioresistance by promoting the transcription of many genes essential for cell survival, DNA repair, and CSCs. Together, these signaling pathways conjointly protect cancer cells from radiation injury and promote radioresistance (Figure 8).

## Data Availability

Not applicable.
